# Clinicopathological, therapeutic and prognostic features of the triple-negative tumors in moroccan breast cancer patients (experience of Hassan II university hospital in Fez)

**DOI:** 10.1186/1756-0500-4-500

**Published:** 2011-11-16

**Authors:** Yousra Akasbi, Sanae Bennis, Fouad Abbass, Kawtar Znati, Khalid Amrani Joutei, Afaf Amarti, Omar EL Mesbahi

**Affiliations:** 1 Medical Oncology unit, Hassan II University Hospital, 19 Rue Jebel Zalagh 2 Narjiss C, 30006 Fez, Morocco; 2 Laboratory Biology of cancers-Faculty of Medicine & Pharmacy, Fez, Morocco; 3 Department of Pathology, Hassan II University Hospital, Fez, Morocco; 4 Laboratory of Bioactive Molecules, Faculty of Sciences & Technology, Fez, Morocco

**Keywords:** Triple negative breast cancer, Clinico-pathological, Prognostic features

## Abstract

**Introduction:**

Triple-negative breast cancer (TNBC) is defined as a group of breast carcinomas that are negative for expression of hormone receptors (ER, PR) and Her2, we can distinguish between two groups: basal-like (ER-, PR-, Her2-, cytokeratin (CK) 5/6+ and/or Her1+) and unclassified subtype (ER-, PR-, Her2-, Her1- and CK5/6-).

The aim of this study is to determine the clinicopathological, histological, therapeutic and prognostic features associated with this type of breast cancer.

**Methods:**

This is a retrospective study of 366 female breast cancer patients, diagnosed between January 2007 and June 2010 at the Department of Pathology. Epidemiological, clinical, histological, therapeutic and evolutive data were analyzed. OS and DFS rates were estimated by Kaplan-Meier analysis and a log-rank test to estimate outcome.

**Results:**

A total of 64 women were identified as having TNBC (17.5% of all female breast cancer patients), 12.6% were basal-like, 4.9% were unclassified subtype, with a median age of 45 years. The median histological tumor diameter was 4.3 cm. TNBC were most often associated with a high grade, 49.2% grade III (53% for unclassified subtype, 47.6% for basal-like). Vascular invasion was found in 26.6% of cases (22% for unclassified subtype and 28.3% for basal-like). For the lymph node involvement: 51% had positive lymph nodes, and 22.4% had distant metastases. Neoadjuvant chemotherapy was administered to 18% patients with 26% of complete pathologic response; therefore adjuvant chemotherapy was given to 82%. 98% received anthracycline based regimen and only 30% received taxanes.

The Kaplan-Meier curves based showed the lowest survival probability at 3-years (49% of OS, and 39% of DFS).

**Conclusion:**

TNBC is associated with young age, high grade tumors, advanced stage at diagnosis, difference chemo response compared to other subtypes, and shortest survival. Critical to optimal future management is accurate identification of truly triple negative disease, and adequately powered prospective TNBC trials to establish treatment efficacy and define predictive biomarkers.

## Background

Breast cancer is a heterogeneous disease with different morphologies, molecular profiles, clinical behavior and response to therapy.

Triple-negative breast cancer (TNBC) is defined as a group of breast carcinomas that are negative for expression of hormone receptors (ER, PR) and HER2, we can distinguish between two groups: basal-like (ER-, PR-, Her2-, cytokeratin (CK) 5/6+ and/or Her11+) and unclassified subtype (ER-, PR-, Her2-, Her1- and CK5/6-). These patients with triple-negative tumors have a relatively poor outcome and cannot be treated with endocrine therapy or therapies targeted to human epidermal growth factor receptor type 2 (HER2), in contrast with hormonal receptor positive and HER2+ breast cancers.

Given these characteristics, triple-negative breast cancer is a challenge in today's clinical practice.

The aim of this study is to determine the clinico-pathological, histological, therapeutic and prognostic features associated with this type of breast cancer.

It is critical to recognise that TNBC is a heterogeneous disease, for which chemotherapy alone is inadequate for the majority of patients. New treatment options are urgently required.

## Methods

This is a retrospective study of 366 female breast cancer patients diagnosed between January 2007 and June 2010 at the Department of Pathology, Hassan II University Hospital.

Epidemiological, clinical, histological, therapeutic and evolutive data were analyzed.

The histological classification was based on the criteria set by the World Health Organization. The histological grade is based on the Scarff-Bloom-Richardson grading system (SBR). It is based on a combined score for nuclear grade, mitotic rate, and histologic grade or architectural differentiation. Each characteristic is given a score of 1 to 3, resulting in a total score ranging from 3 to 9. Grade 1 includes tumors with combined scores of 3, 4 or 5; grade 2 includes scores of 6 and 7; and grade 3 includes tumors with the scores of 8 and 9.

Her2 immunohistochemical was carried out using the HercepTest. Her2 score 2+ cases were analysed and completed by FISH Method. They were performed using the PathVysion HER2 DNA Probe (Abbott Vysis Inc., Downers Grove, IL) according to the manufacturer's protocol. The probe cocktail, including the LSI HER-2/neu probe and the CEP17 probe. Fluorescence signals were analyzed and digitalized using the CytoVision™ image analysis system (Applied Imaging International Ltd., Newcastle-Upon-Tyne, UK). Between 60 and 100 nuclei were scored from each case. Signal ratios (HER2:CEP17) of ≥ 2.2 were classified as amplified. In the absence of positive FISH data, tumors scored 2+ by IHC were considered negative for HER-2.

OS and DFS rates were estimated by Kaplan-Meier analysis and a log-rank test to estimate the outcome. OS was determined as the length of time from the date of surgery until either the date of death (from any cause) or the date of last follow-up. DFS was defined as the length of time from the date of surgery to any relapse or death.

Consent was obtained from the participants in this research.

Ethical approval was not obtained in our research.

## Results

A total of 64 patients with breast cancer, were identified as having triple-negative breast cancer (17.5%), which 12.6% were basal-like (CK5/6 positive and or Her1 positives), 4.9% were unclassified subtype (CK5/6 and Her1 negatives), a median age and histological tumor diameter were respectively 45 years and 4.3 cm (Table 1).

**Table 1:** Description of the characteristics in the population study

Characteristics	%
Median age	45 years

Median histological tumor diameter	4.3 cm

Triple-negative breast cancer	17.5%

Basal-like	12.6%

Unclassified subtype	4.9%

Histological type	

Invasive ductal carcinomas	78%

Metaplasic carcinoma	9.4%

Medullar carcinoma	6.3%

Other types	6.3%

Histological grade SBR	

I	3.3%

II	47.5%

III	49.2%

Vascular invasion	26.6%

Positive lymph nodes	51%

Distant metastases	22.4%

AJCC staging	

stage I	17.2%

stage IIA	20.7%

stage IIB	13.8%

stage IIIA	10.3%

stage IIIB	15.5%

metastatic	22.4%

Treatment modalities	

Surgery	94%

Radical mastectomy	40%

Conservative surgery	60%

Chemotherapy	

Neoadjuvant chemotherapy	18%

Adjuvant chemotherapy	82%.

Anthracycline based regimen	98%

Only taxanes	30%

Complete pathologic response	26%

Outcome	

Overall survival at 3 years	49%

Disease-free survival at 3 years	39%

TNBC were most often associated with invasive ductal carcinomas (78%), and a high grade, 49.2% grade III (53% for unclassified subtype, 47.6% for basal-like), 47.5% grade II. However, 3.3% triple-negative breast cancer were grade I (0% et 4.8% unclassified subtype and basal-like respectively). A small percentage of cancer was diagnosed metaplasic carcinoma (9.4%) or medullar carcinoma (6.3%).

Vascular invasion was found in 26.6% of cases (22% for unclassified subtype and 28.3% for basal-like subtype).

For the lymph node involvement: 51% had positive lymph nodes, and 22.4% had distant metastases. For the AJCC staging 17.2% were classified stage I, 20.7% stage IIA, 13.8% stage IIB, 10.3% stage IIIA, 15.5% stage IIIB, and 22.4% were metastatic.

For treatment modalities, 94% underwent surgery (radical mastectomy in 40% of cases and 60% had conservative surgery). Neoadjuvant chemotherapy was administered to 18% patients with 26% of complete pathologic response; therefore adjuvant chemotherapy was given to 82%. 98% received anthracycline based regimen and only 30% received taxanes.

**Figure 1:**
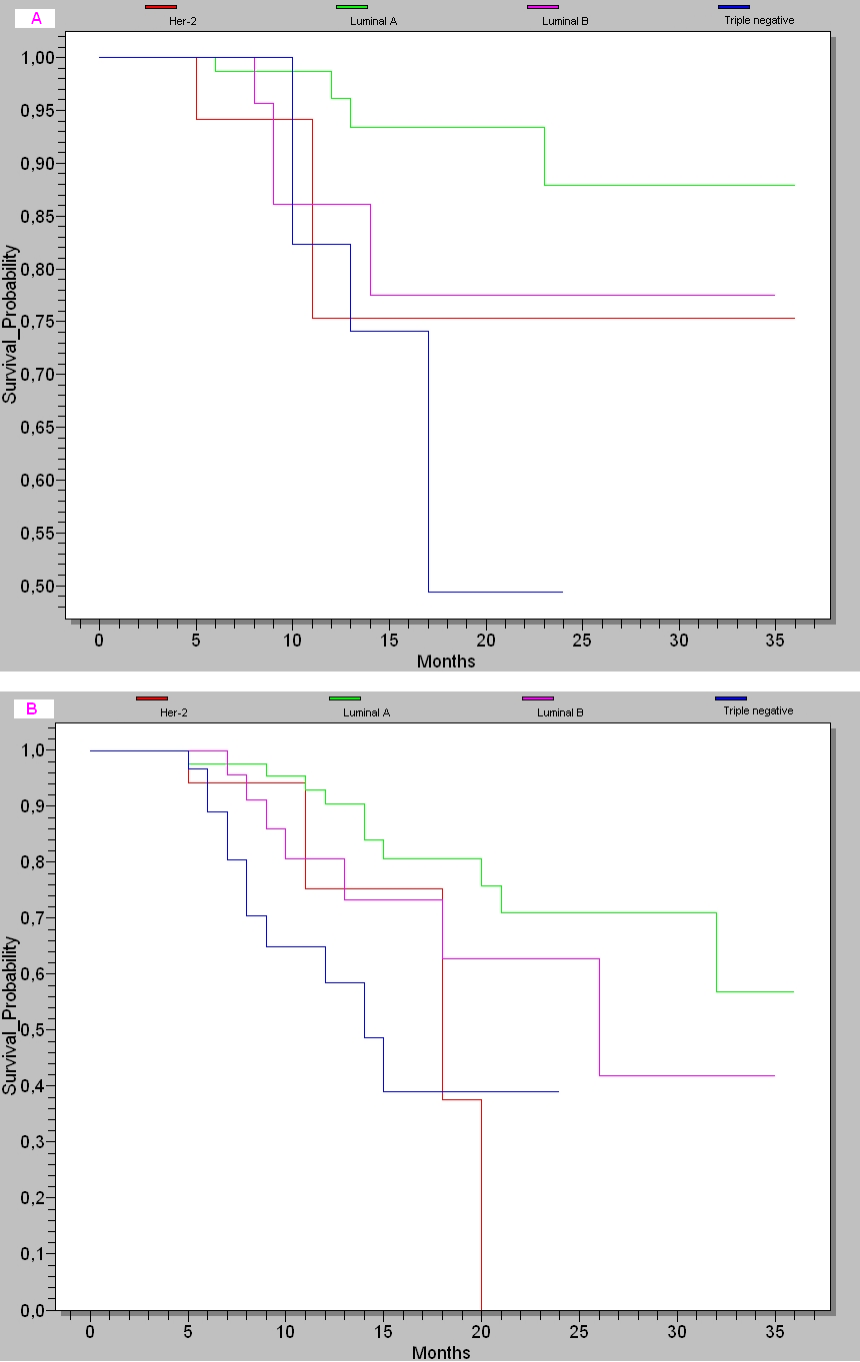
**a Kaplan-Meier curve illustrating the disease-free survival at 3 years of follow-up**. **b **Kaplan-Meier curve illustrating the overall survival at 3 years of follow-up.

The Kaplan-Meier curves based showed the lowest survival probability at 3-years (49% of OS, and 39% of DFS) (Figure 1).

## Discussion

A Pub Med search of the medical literature shows that the first mention of "triple-negative" breast cancer was in October 2005; since then, the term has appeared in more than 600 publications [1]. This increase reflects the growing recognition of the importance of TNBC by oncologists, biologists, pathologists, and geneticists, as well as by approximately 10-17% of women identified with breast cancer.

TNBC are defined as tumors that lack expression of estrogen receptor (ER), progesterone receptor (PR), and HER2.

Among the five intrinsic subgroups of breast cancer, we identified basal like breast cancer by expression genes profiling revealed by microarray test [2]. This subgroup is characterized by an absence or low levels of expression of ER, an absence of HER2, and expression of genes usually found in basal or myoepithelial cells of the normal breast (CK5/6+, Her1+) [2,3].

In this study, we have used the same definition of TNBC.

TNBC generally occurs in younger women, less than 50 years, and is associated with a high risk of distant recurrence and death, especially during the first 3-5 years of follow up [4,5]. Our series report the same results, the TNBC group is associated with the high percentage of death (47%) and 64%of patients were aged less than 50 years.

Some risk factors for developing basal-like breast cancers have been identified [6,7]. The association between *BRCA1 *mutations and the development of TNBC is well established [8]. A recent report suggests that *BRCA1 *mutations occur in close to 20% of sporadic TNBC and are associated with improved prognosis [9]. It is currently unknown whether *BRCA1*-mutated tumors more closely resemble claudin-low or basal-like TNBC. Moreover, tumors arising in *BRCA1 *mutation carriers illustrate sensitivity to poly-(ADP)-ribose polymerase inhibitors, thus suggesting that mutated *BRCA1 *within TNBC could be predictive of response to this novel class of agents [10,11].

In the Carolina Breast Cancer Study, 20% of the tumors were basal-like. In our series, 13% of the tumors were basal-like. Our results demonstrated that the basal-like subtype was most common among premenopausal women (61%) compared with postmenopausal (39%) similar to that reported by other series [12,13]. Interestingly, a recent study of risk among different racial and ethnic subtypes in the Women's Health Initiative suggested that among African American women, traditional risk factors such as menstrual and pregnancy history, body mass index, and activity failed to explain differences in incidence from white women [14].

The grade III was the most common as in our series, corresponding to 53% in unclassified subtype, 48% in basal-like, 48% in Her2-overexpressing, 28% in luminal B, 23% in luminal A (*p = 0,005*), which concord with other studies such as Livasy et al. [15] who demonstrated that the most consistent pathologic features for basal-like subtype including high grade, high mitotic count, geographic necrosis, pushing border, prominent lymphocytic infiltrates, solid growth pattern, central fibrotic/acellular area, and less association with ductal carcinoma in situ.

Carey et al. [13] showed that a high mitotic index was seen in 87% of basal-like subtype, 69% in the Her2-overexpressing subtype, and only 31% and 32% in luminal A and B subtypes, respectively. Secondly they showed a marginally significant difference (*p *= 0.04) in the rate of lymph node involvement, with the highest being among Her2-overexpressing subtype (56%), followed by luminal B (47%), basal-like (41%), and luminal A (34%). Our series showed a non significant difference (*p = 0,555*) in the rate of the lymph node involvement, with the highest being among luminal B subtype (74%), followed by Her2-overexperssing (66%), luminal A (63.5%), unclassified (61.5%), and basal-like (55%).

The basal-like and Her2-overexpressing subtypes have the shortest survival, luminal A has the longest survival, and luminal B has an intermediated prognosis [16]. These results are similar to our series. The Kaplan-Meier curves based on the subclasses from Figure 1 showed a highly significant difference in OS at 3-years between the subtypes (Figure 1a, Log-Rank test: *p *= 0,042), with the triple negative subtype associated with the lowest survival probability (52%), but the luminal A was associated with the best survival probability (88%) compared to those in the other subtypes (77% for luminal B, 75% for Her2-overexpressing). These subtypes also differed significantly in 3-years DFS (Figure 1b, Log-Rank test: *p *= 0.002): luminal A (59%), luminal B (41%), Her2-overexpressing (38%) and triple negative (39%). Breast cancer patients with TNBC tumors had a poorer prognosis in terms of DFS and OS than those with luminal A tumors in the present study as previously indicated in the report by the most studies. Although some reports suggest that they respond to chemotherapy better than other types of breast cancer, prognosis remains poor.

This is due to poor disease-free intervals in the adjuvant and neoadjuvant setting, shortened progression-free survival associated to a more aggressive clinical course in the metastatic setting, and the lack of targeted therapy.

TNBC have a good initial response to chemotherapy, particularly anthracycline and taxanebased therapy. Although these tumors are initially sensitive to standard neoadjuvant chemotherapy, they continue to exhibit a short disease-free survival [17].

Rouzier et al. [18] evaluated complete pathologic response with standardized neoadjuvant therapy among 82 patients, and found that the complete pathologic response rates were 45% for basal-like and Her2-overexpressing subtypes and only 6% for luminal subtypes

Goldstein et al. [19] also demonstrated that there is differential chemoresponse in different subtypes, with basal-like and Her2-overexpressing subtype being more sensitive, luminal A being more resistant, and luminal B being intermediate.

Carey et al. showed that basal-like and Her2-overexpressing subtypes are more sensitive to anthracycline-based neoadjuvant chemotherapy compared with luminal breast cancer [20].

Recently published neoadjuvant studies have clarified the fact that patients who have a good pathologic outcome from surgery also have a good clinical response. However, within the group of patients who have residual disease after completing neoadjuvant chemotherapy, a poorer prognosis is seen in the triple-negative subgroup [20].

But in our series, after neoadjuvant chemotherapy based on anthracyclines and taxanes, complete pathologic response was estimated as 62% in luminal A, 26% in triple negative breast cancer, and 12% in Her2-overexpressing subtype. The few percentage of complete pathologic response in Her2-overexpressing was explained by the lack of availability of this drug in the hospital during the study period.

Currently, a major challenge is to identify target molecules for basal-like subtype. Some of the possible molecular targets for basal tumors that have been proposed include EGFR and vascular endothelial growth factor [21]. The c-kit, overexpressed in up to 31% of basal-like subtype, seems not to be a suitable target at present, because it lacks the activated mutation in breast cancer that conveys sensitivity to imatinib [22].

The lack of therapeutic effect of imatinib was shown in a phase II study of unselected patients with metastatic breast cancers [23]. To complicate the matter even further, a recent report has suggested, that the same chemotherapeutic agents may have different mechanisms of action indifferent subtypes of breast cancers [24].

X-linked inhibitor of apoptosis protein (XIAP) is a viable novel biomarker and prognostic factor for invasive ductal breast cancer with triple-negative phenotype. The expression of XIAP is significantly correlated with a more aggressive tumor phenotype and decreased OS and DFS [25].

## Conclusion

Triple-negative breast cancer is associated with young age, high grade tumors, advanced stage at diagnosis, difference chemo response compared to other subtypes, and shortest survival.

Critical to optimal future management are accurate identification of truly triple negative disease and adequately powered prospective TNBC trials to establish treatment efficacy and define predictive biomarkers.

## List of abbreviations used

CK: Cytokeratin; DFS: Disease free survival; EGFR: Epidermal growth factor receptor; ER: Estrogen receptor; FISH: Fluorescence in situ hybridization; HER2: Human epidermal growth factor receptor type 2; IHC: Immunohistochemical; PR: Progesterone receptor; OS: Overall survival; SBR: Scarff-bloom-richardson grading system; TNBC: Triple-negative breast cancer; XIAP: X-linked inhibitor of apoptosis protein.

## Competing interests

The authors declare that they have no competing interests.

## Authors' contributions

All authors contributed to the interpretation of the data and were responsible for reviewing the manuscript. All authors have read and approved the final manuscript.
